# Supplementary material for: Genetic Characterization of Non-Lymphogranuloma venereum Chlamydia trachomatis Indicates Distinct Infection Transmission Networks in Spain
Source: Int J Mol Sci. 2023 Apr 8;24(8):6941. doi: 10.3390/ijms24086941 (PMC10138622; doi:10.3390/ijms24086941)
Supplement: Supplementary file 1 [file ijms-24-06941-s001.zip › ijms-2258635-supplementary.pdf]

Supplementary Figure S1. Map with the seven hospitals in Spain participating in this study: Asturias University Hospital (Oviedo, Asturias), Cabueñes University Hospital (Gijón, Asturias), Donostia University Hospital (San Sebastián, Basque Country), Vall d´Hebrón University Hospital (Barcelona, Catalonia), Miguel Servet University Hospital (Zaragoza, Aragón), Son Espases University Hospital (Palma de Mallorca, Balearic Islands), and Valme Univeristy Hospital (Seville, Andalucia).

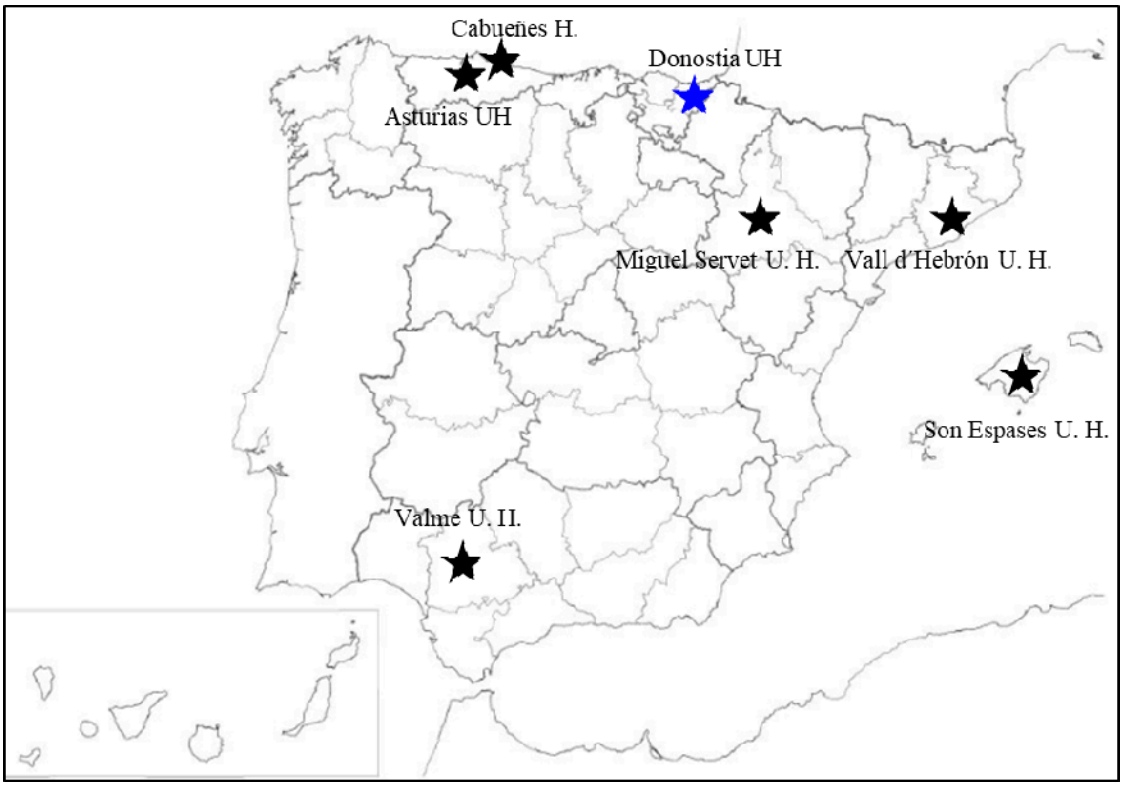

Star in blue: laboratory where *ompA* genotyping and sequenciotyping was performed.

Supplementary Figure S2. Pie charts of the 698 cases of *Chlamydia trachomatis* infection in Spain (2018-2019) included in the study by a) geographical area; b) anatomical site of sampling; c) age group (years); d) sex; e) sexual behaviour; f) clinical manifestations; and g) place of origin.

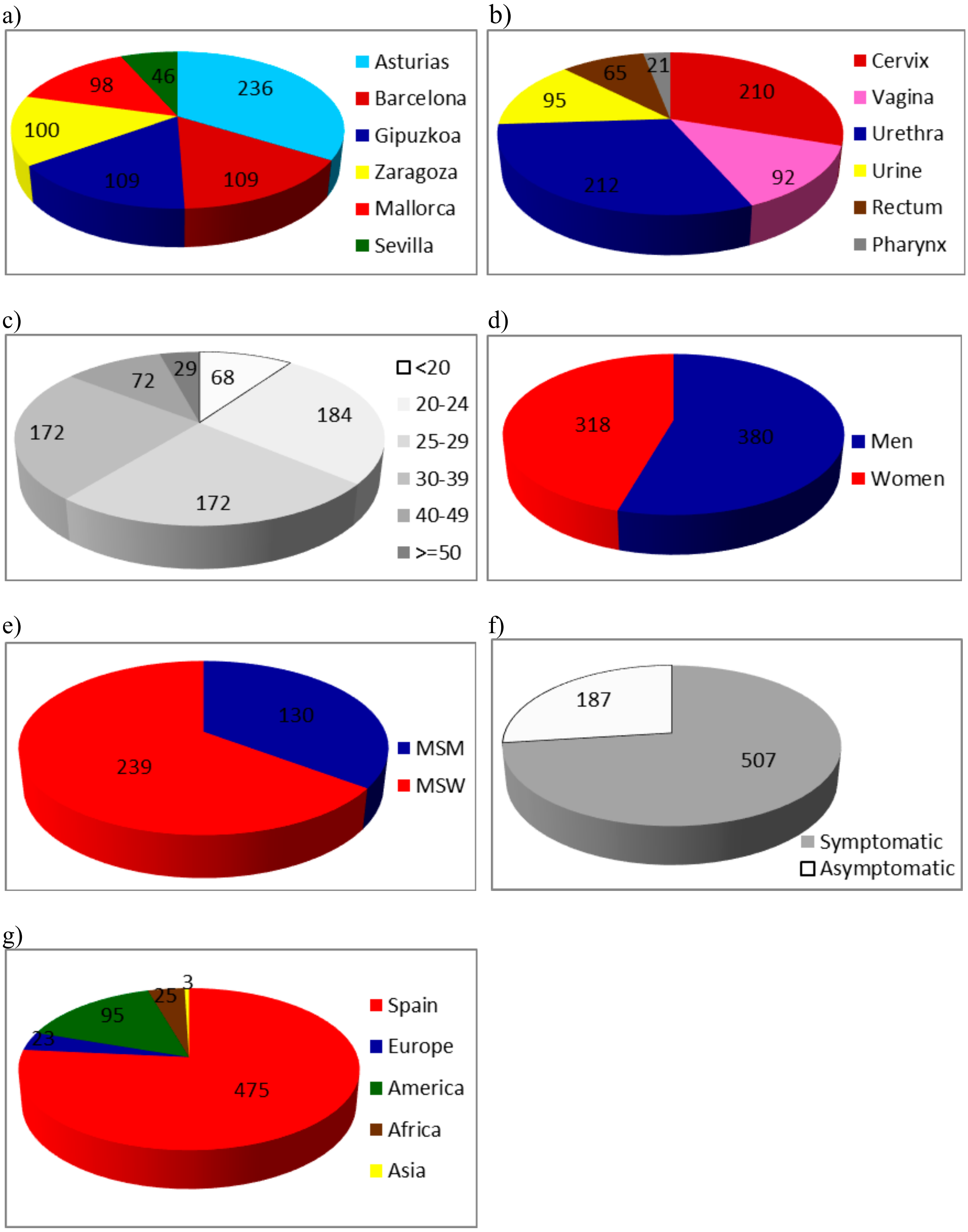

MSW: men who have sex with women; MSM: men who have sex with men.

Supplementary Table S1. Distribution of sequence types (STs) by genotype of *Chlamydia trachomatis* in 158 cases of this infection in Spain (2018-2019).

MLST (n)/genotype	E	D	F	G	J	I	H	K	B	Total	(%)
3	2									2	1.3
12		1	2							3	1.9
27,265,279,301,575,578,582				1x7						7	4.4
28				2						2	1.3
**30**								4		4	2.5
**33**				5						5	3.2
34,358,577,579								1x4		4	2.5
**35**		6								6	3.8
**52**				10						10	6.3
56	1			1						2	1.3
58		2								2	1.3
**59**	3									3	1.9
64,147,154,573,586	1x5									5	3.2
85,110,231,572			1x4							4	2.5
**90**		1	3							4	2.5
**97**							6			6	3.8
**100**						6				6	3.8
104					2					2	1.3
**108**					5				1	6	3.8
**109**		4								4	2.5
112,157,267,583,584,588					1x6					6	3.8
128				3						3	1.9
130,517,581						1x3				3	1.9
136					2					2	1.3
**148**			5							5	3.2
194,243,420,585,587		1x5								5	3.2
208									1	1	0.6
274						2				2	1.3
275				1		1				2	1.3
**276**						7				7	4.4
**327**	3									3	1.9
380								2		2	1.3
443,574,580,590							1x4			4	2.5
497	2									2	1.3
571		4								4	2.5
576					2					2	1.3
589				2						2	1.3
Not able to be typed	*2*	*5*	*1*	*3*	*3*		*1*		*1*	*16*	*10.1*
Total (%)	18 (11.4)	28 (17.7)	15 (9.5)	34 (21.5)	20 (12.7)	19 (12.0)	11 (7.0)	10 (6.3)	3 (1.9)	**158**	100.0
Genotyped, n (%)	225 (35.4)	119 (18.7)	106 (16.7)	70 (11.0)	47 (7.4)	40 (6.3)	13 (2.0)	13 (2.0)	3 (0.5)	636	

Sequence typed, %	8.0	23.5	14.2	48.6	42.6	47.5	84.6	76.9	100	24.8	
STs/genotype	10/16	11/23	7/14	14/31	10/17	7/19	5/10	6/10	2/2	67/142	vs 9/636
(% diversity)	(62.5)	(47.8)	(50.0)	(45.2)	(58.8)	(36.8)	(50)	(60)	(100)	(47.2)	(1.4)

In bold, the most common STs; in blue, STs found in more than one genotype.

Supplementary Table S2. Distribution of sequence types (STs) by genotype of *Chlamydia trachomatis*, sex and sexual behaviour in 142 cases of this infection in Spain (2018-2019).

Genotype	ST	Woman	Man	MSW	MSM	n/ST
E	**59**	1	2	1	1	3
	**327**	0	3	3	0	3
	3	0	2	1	1	2
	497	1	1	1	0	2
	56	0	1	0	1	1
	64	1	0	0	0	1
	147	1	0	0	0	1
	154	0	1	0	1	1
	573	1	0	0	0	1
	586	1	0	0	0	1
D	**35**	4	2	1	1	6
	**109**	0	4	1	3	4
	**571**	0	4	0	4	4
	58	0	2	0	2	2
	12	1	0	0	0	1
	90	0	1	0	1	1
	194	0	1	0	1	1
	243	0	1	0	1	1
	420	0	1	1	0	1
	585	1	0	0	0	1
	587	0	1	1	0	1
F	**148**	2	3	3	0	5
	**90**	1	2	2	0	3
	12	1	1	0	1	2
	85	0	1	1	0	1
	110	1	0	0	0	1
	231	0	1	0	1	1
	572	0	1	0	1	1
G	**52**	1	9	1	8	10
	**33**	0	5	1	4	5
	128	3	0	0	0	3
	28	2	0	0	0	2
	589	0	2	0	2	2
	27	1	0	0	0	1
	56	1	0	0	0	1
	265	1	0	0	0	1
	275	1	0	0	0	1
	279	1	0	0	0	1
	301	0	1	0	1	1
	575	1	0	0	0	1
	578	0	1	1	0	1
	582	0	1	0	1	1
J	**108**	0	5	0	5	5
	104	1	1	1	0	2
	136	2	0	0	0	2
	576	1	1	1	0	2
	112	0	1	0	1	1
	157	1	0	0	0	1
	267	0	1	1	0	1
	583	0	1	0	1	1
	584	0	1	0	1	1
	588	1	0	0	0	1
I	**276**	4	3	3	0	7

	**100**	5	1	1	0	6
	274	1	1	1	0	2
	130	0	1	0	1	1
	275	1	0	0	0	1
	517	1	0	0	0	1
	581	1	0	0	0	1
H	**97**	3	3	2	1	6
	443	0	1	1	0	1
	574	1	0	0	0	1
	580	1	0	0	0	1
	590	1	0	0	0	1
K	**30**	1	3	3	0	4
	380	2	0	0	0	2
	34	1	0	0	0	1
	358	1	0	0	0	1
	577	0	1	1	0	1
	579	0	1	0	1	1
B	108	0	1	0	1	1
	208	0	1	1	0	1
**Total**		59	83	35	48	**142**
Different STs, n (%)		39 (66.1)	43 (51.8)	25 (71.4)	26 (54.2)	67/142 (47.2)

MSW: men who have sex with women; MSM: men who have sex with men.
In bold, the most common STs; in blue, STs found in more than one genotype.
